# Joint Torque Reduction of a Three Dimensional Redundant Planar Manipulator

**DOI:** 10.3390/s120606869

**Published:** 2012-05-25

**Authors:** Samer Yahya, Mahmoud Moghavvemi, Haider Abbas F. Almurib

**Affiliations:** 1 Center of Research in Applied Electronics (CRAE), University of Malaya, Kuala Lumpur 50603, Malaysia; E-Mail: mahmoud@um.edu.my; 2 Faculty of Electrical and Computer Engineering, University of Tehran, P.O. Box 14399-57131, Tehran, Iran; 3 Department of Electrical & Electronic Engineering, University of Nottingham Malaysia, Jalan Broga, Semenyih 43500, Malaysia; E-Mail: haider.abbas@nottingham.edu.my

**Keywords:** redundant manipulator, dynamics, robot, rotary encoders, joint torques reduction

## Abstract

Research on joint torque reduction in robot manipulators has received considerable attention in recent years. Minimizing the computational complexity of torque optimization and the ability to calculate the magnitude of the joint torque accurately will result in a safe operation without overloading the joint actuators. This paper presents a mechanical design for a three dimensional planar redundant manipulator with the advantage of the reduction in the number of motors needed to control the joint angle, leading to a decrease in the weight of the manipulator. Many efforts have been focused on decreasing the weight of manipulators, such as using lightweight joints design or setting the actuators at the base of the manipulator and using tendons for the transmission of power to these joints. By using the design of this paper, only three motors are needed to control any n degrees of freedom in a three dimensional planar redundant manipulator instead of n motors. Therefore this design is very effective to decrease the weight of the manipulator as well as the number of motors needed to control the manipulator. In this paper, the torque of all the joints are calculated for the proposed manipulator (with three motors) and the conventional three dimensional planar manipulator (with one motor for each degree of freedom) to show the effectiveness of the proposed manipulator for decreasing the weight of the manipulator and minimizing driving joint torques.

## 1. Introduction

Theoretically, for a structure of the robot manipulator one actuator can be mounted on each link to drive the next link via a speed reduction unit, but actuators and speed reducers installed on the distal end become the load for actuators installed on the proximal end of a manipulator, resulting in a bulky and heavy system [1]. To reduce the weight and the inertia of a robot manipulator, many mechanisms have been proposed so far to remove the weight restriction. Some reported by [2,3] include:

- Lightweight joint design based on a special rotary joint [4–6]
- Provision of a powerful slider at the base to bear as much required driving force as possible [7]
- The parallel mechanism is another method to reduce the mass and inertia of the manipulator [8]. A typical parallel manipulator consists of a moving platform that is connected with a fixed base by several limbs. Generally, the number of degrees of freedom of a parallel manipulator is equal to the number of its limbs. The actuators are usually mounted on or near the base, which contributes to reduce the inertia of manipulators, and
- Concentration of the actuators at the base and transmission of the power to each joint through tendons or a special transmission mechanism [2,3,9]. This mechanism allows the actuators to be situated remotely on the manipulator base, allowing the manipulator to be made more lightweight and compact.

For a serial manipulator, direct kinematics are fairly straightforward, whereas inverse kinematics becomes very difficult. Reference [10] proposes a fused smart sensor network to estimate the forward kinematics of an industrial robot, while reference [11] measures the range data with respect to the robot base frame using the robot forward kinematics and the optical triangulation principle. The inverse kinematics problem is much more interesting and its solution is more useful, but one of the difficulties of inverse kinematics is that when a manipulator is redundant, it is anticipated that the inverse kinematics has an infinite number of solutions. This implies that, for a given location of the manipulator's end-effector, it is possible to induce a self-motion of the structure without changing the location of the end-effector. In this paper we depend on our prior works [12,13] which present a new method to solve the problem of multi-solutions of a three dimensinal planar redundant manipulator. Because this paper explains the dynamic of the manipulator and not its kinematics, the inverse kinematics methods will not be explained here. For more details about the inverse kinematics of redundant manipulators, our works [14–16] can be checked.

It is mentioned earlier that the proposed manipulator could be used to reduce the weight of the manipulator which yields to a decrease in the size (power) of the motors used to control the manipulator. To show the effectiveness of the proposed manipulator in reducing the torques of its motors the inverse dynamic of the manipulator has been calculated mathematically. The inverse dynamic model provides the joint torques in terms of the joint positions, velocities and accelerations. For robot design, the inverse dynamic model is used to compute the actuator torques, which are needed to achive a desired motion [17]. Several approaches have been proposed to model the dynamics of robots. The most frequently employed in robotics are the Lagrange formulation and the Newton-Euler formulation. Because the Lagrange formulation is conceptually simple and systematic [18], it has been used in this paper. The Lagrange formulation provides a description of the relationship between the joint actuator forces and the motion of the mechanism, and fundamentally operates on the kinetic and potential energy in the system [19].

The work presented in this paper is based on our previous work [14], which presents a mechanical design for a three dimensional planar redundant manipulator, which guarantees to decrease the weight of the manipulator by decreasing the number of motors needed to control it. Because the inverse kinematics model gives an infinite number of solutions for a redundant manipulator, consequently, secondary performance criteria can be optimized [17], such as avoiding singular configurations and minimizing driving joint torques. Reference [14] studied the kinematics of the manipulator of this paper and showed in details its ability to avoid singular configurations. A comparison of the manipulability index values and the manipulability ellipsoids for the manipulator is made with the manipulability index values and the manipulability ellipsoids of the PUMA arm to show the effectiveness of using the proposed manipulator to avoid singularity. In this paper, the dynamics of this manipulator are explained in detail. The contribution of this work is to explain the ability of this manipulator for joint torque minimization. The links and motors mass distribution is studied for both the proposed (with three motors) and conventional manipulators (six motors). The driving joint torques have been studied for the proposed manipulator for each joint and the results are compared with the results of the conventional manipulators to show the effectiveness of this manipulator for minimizing driving joint torques.

## 2. The Mechanical Design of the Manipulator

**Figure 1.**
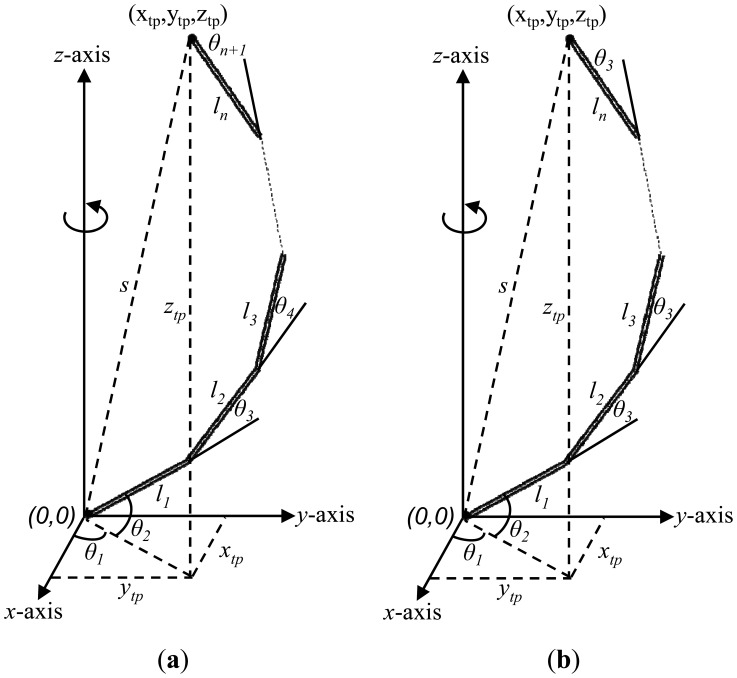
(**a**) A three dimensional planar redundant manipulator configuration; (**b**) A three dimensional planar redundant manipulator configuration using the method of [12,13].

To control the motion of the end-effector of the manipulator shown of Figure 1(a), all the motors of the manipulator should be controlled. For example, to control a five links planar redundant manipulator with the ability to rotate the entire manipulator around its vertical axis, the six motors (five motors for each joint angle and one motor to rotate the entire manipulator around its vertical axis) of the manipulator should be controlled. Using the method of our papers [12,13], the configuration of the manipulator will have three angles to be controlled instead of *n* angles. Figure 1(b) shows the configuration of the manipulator when there are just three angles that need to be controlled.

**Figure 2.**
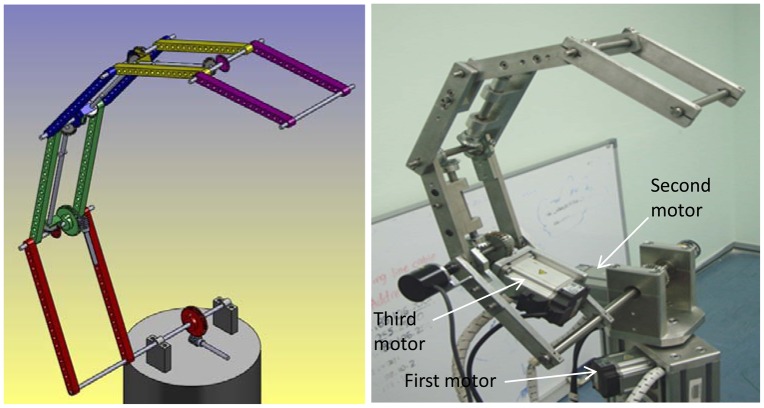
The manipulator used in experiments [14]. The draft of the manipulator using the SolidWorks software (**left**). The mechanical design of the manipulator (**right**).

Because the end-effector can follow any desired path by controlling three angles (*θ_1_*, *θ_2_* and *θ_3_*) only, therefore instead of using a motor for each joint angle, three motors can be used for controlling the manipulator. This means that for any number of degrees of freedom three dimensional planar redundant manipulators, the weight of the links will be significantly decreased using the proposed design. To make the manipulator capable of moving in a three dimensional work space, one motor will control the value of *θ_1_*—this means controlling the rotation of the entire manipulator around the vertical axis. This motor is situated in such a way as to rotate the base of the manipulator around the *z*-axis. The second motor controls the value of *θ_2_*, which means the rotation of the entire manipulator with its configuration. The motor is situated at the base. The third motor controls the value of *θ_3_* and this motor is situated on the first link. This motor will rotate the second link of manipulator about the second axis, and because all the next links should rotate about their axes by the same angle *θ_3_* therefore, there is no need to use motor for each joint angle, but the rotation of the second motor will be transferred to the next joints using gears boxes. Figure 2 shows the mechanism of the proposed manipulator.

**Figure 3.**
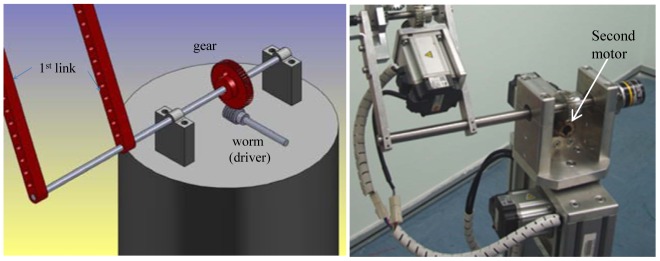
The design of the second joint angle (first link with second motor) of the manipulator [14]. The draft of the second joint angle using the SolidWorks software (**left**). The mechanical design of the second joint angle (**right**).

Elaborating further, the second motor is connected to the first link using a worm gear to control the angle *θ_2_*. Figure 3 shows the position of the second motor.

**Figure 4.**
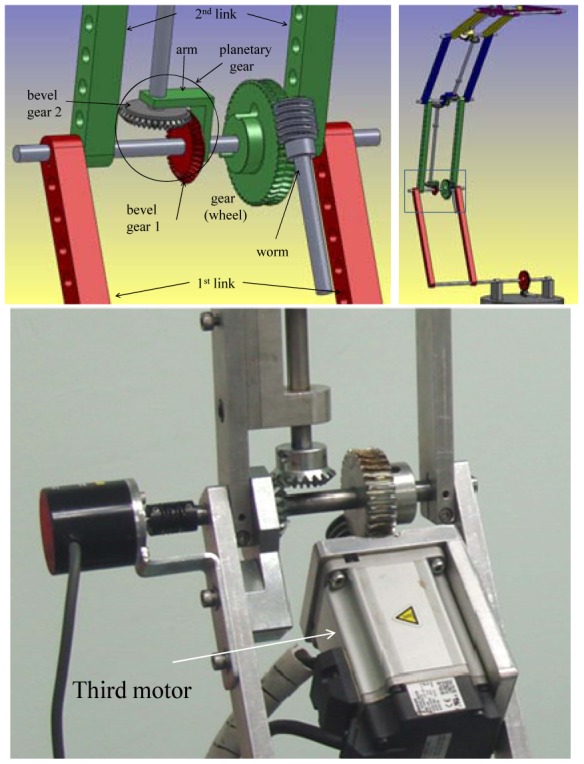
The design of the third joint angle (second link with third motor) of the manipulator [14]. The draft of the third joint angle using the SolidWorks software (**top left**). The draft of the whole manipulator using the SolidWorks software (**top right**). The mechanical design of the third joint angle (**bottom**).

The third motor is connected to the second link using a worm gear for the same reasons it was used with the first link. Controlling the third motor means controlling the angle between the first link and the second link *i.e.*, the angle *θ_3_*. Figure 4 shows the position of the third motor.

**Figure 5.**
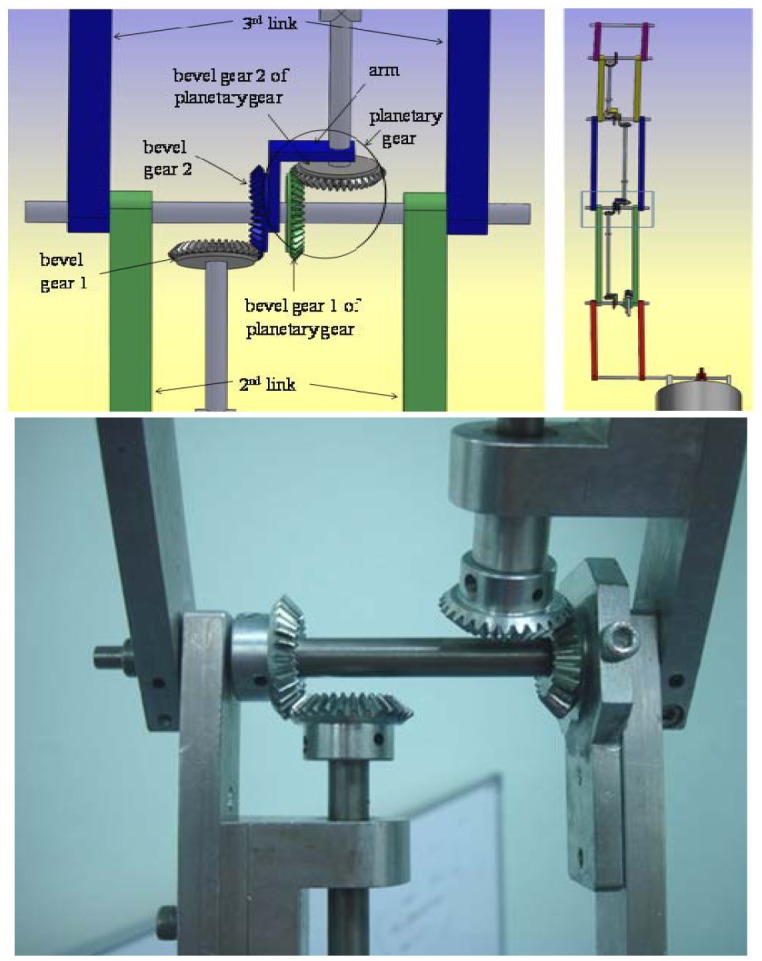
The design of the fourth joint angle (third link) of the manipulator [14]. The draft of the fourth joint angle using the SolidWorks software (**top left**). The draft of the whole manipulator using the SolidWorks software (**top right**). The mechanical design of the fourth joint angle (**bottom**).

**Figure 6.**
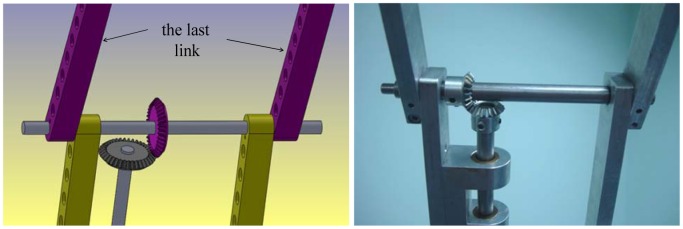
The last joint of the manipulator.

The mechanism of the third link is shown in Figure 5. The same mechanism of the second link is used; the only one difference is that instead of using s worm as a driver and s wheel gear as a driven, two bevel gears are used. The same mechanism of the third link can be used with the next links. The last link has the mechanism shown in the Figure 6. For further details of the mechanical design of the manipulator, our reference [14] can be checked.

To ensure that all the links move at the same joint angle, the ratio between the bevel gears of each planetary gear should be equal to one. This means the bevel gears of each planetary gear should have the same diameter and number of teeth. If this arm is fixed, we get:

(1) $$\frac{w_{1}}{w_{2}}=-\frac{N_{2}}{N_{1}}$$

where *w* is the angular velocity of gear and *N* is the number of teeth of gear. In our manipulator, it is noted that the first gear is fixed while the second gear and the arm are rotating. It is desired that both the arm and the second gear have the same angular velocity. Because the arm is not stationary, then we cannot use the previous equation. *i.e.*, the mechanism is not an ordinary gear train but a planetary gear train. To convert this planetary gear train to an ordinary gear train, it is assumed that the arm is stationary while a first gear has an angular velocity and not fixed. This means that:

(2) $$w_{1}^{\prime }=w_{1}-w_{a}$$

(3) $$w_{a}^{\prime }=w_{a}-w_{a}=0$$

And because the second gear will continue rotating with the same angular velocity, then:

(4) $$w_{2}^{\prime }=w_{2}$$

Now the Equation (1) can be rewritten as follows:

(5) $$\frac{w_{1}^{\prime }}{w_{2}^{\prime }}=-\frac{N_{2}}{N_{1}}=\frac{w_{1}-w_{a}}{w_{2}}$$

For our manipulator it is desired to move both the arm and the second gear by the same angular velocity w which means:

(6) $$\begin{array}{c} \frac{(0)-w}{w}=-\frac{N_{2}}{N_{1}}\\ N_{1}=N_{2} \end{array}$$

**Figure 7.**
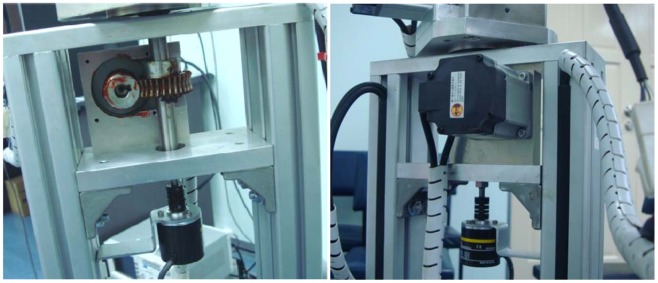
The mechanism of the first motor.

To make the manipulator to have the ability to move in a three dimensional work space, a motor is added to the base of the manipulator to make the whole manipulator capable of rotating around the *z*-axis. This motor controls *θ_1_*. Figure 7 shows the mechanism of the first motor.

**Figure 8.**
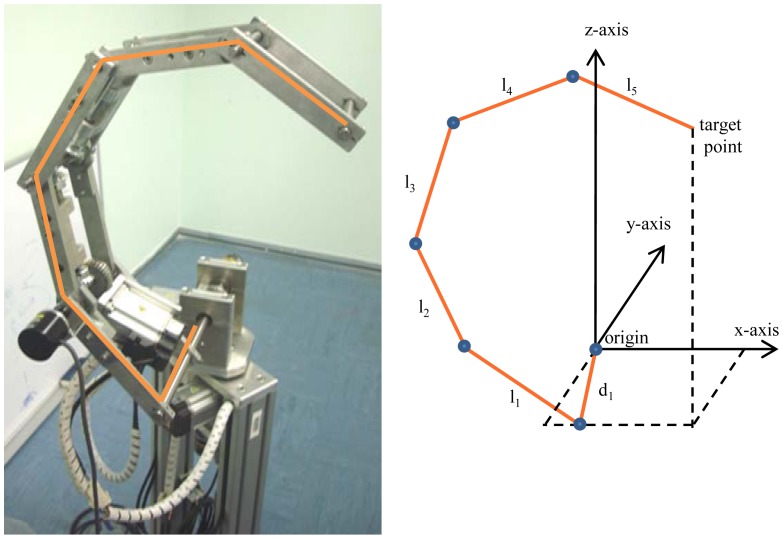
The manipulator used in experiments.

To calculate the transformation matrix of the manipulator, the draft of the manipulator shown in Figure 8, is used. The corresponding link parameters of the manipulator are shown in Table 1. Where *l_1_*, *l_2_*, …, *l_5_* are the length of the links, while *d_1_* is the offset between the origin and the end-effector.

From the links parameters shown in Table 1 and using Equation (7) which defines the transformation matrix *T* for the links [1], we compute the individual transformations for each link:

(7) Ti−1i=[cosθi−sinθicosαisinθisinαiaicosθisinθicosθicosαi−cosθicosαiaisinθi0sinαicosαidi0001]

where *c_i_* = *cos*(*θ_i_*) and *s_i_* = *sin*(*θ_i_*).

(8) T01=[c10s10s10−c1001000001],T12=[c2−s20l1c2s2c20l1s2001d10001],T23=[c3−s30l2c3s3c30l2s300100001]T34=[c4−s40l3c4s4c40l3s400100001],T45=[c5−s50l4c5s5c50l4s500100001],T56=[c6−s60l5c6s6c60l5s600100001]

Finally we obtain the product of all six link transforms:

(9) T06=T01T12T23T34T45T56

## 3. Dynamics of the Manipulator

In this section, the torque of each joint is calculated. To show the effectiveness of the proposed manipulator, the joint torques are calculated using the proposed manipulator (using three motors only) and the conventional manipulators (a motor for each joint).

Let us assume for concreteness that the center of mass of each link is at its geometric center. For the manipulator used in our experiments, the mass of links without the motors are as follow: *ml_1_* = 760 gm, *ml_2_* = 720 gm, *ml_3_* = 680 gm, *ml_4_* = 640 gm, and finally *ml_5_* = 600 gm. These masses are calculated for the manipulator with *l_1_* = 19 cm, *l_2_* = 18 cm, *l_3_* = 17 cm, *l_4_* = 16 cm, *l_5_* = 15 cm and *d_2_* = 21 cm.

**Figure 9.**
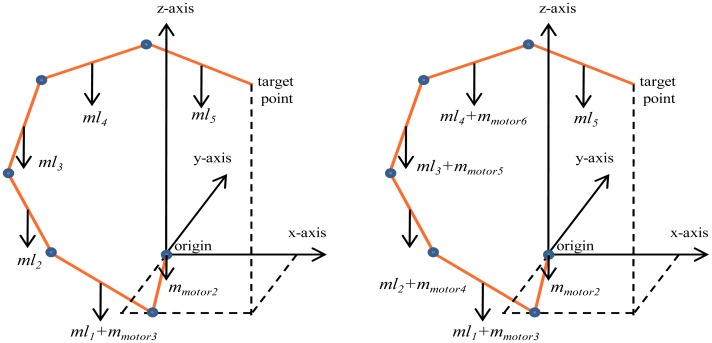
The position of mass for (**a**) the proposed manipulator; (**b**) the conventional manipulator.

The mass of each motor is 1,500 gm; for the manipulator of the proposed design, the first motor and the second motor are located on the base and not on the links themselves. Therefore, for our manipulator, the mass of the first link will be equal to the mass of this link (760 gm) plus the mass of the motor (1,500 gm) that controls the next links. Because there are no more motors, the mass of the links will be: *m_1_* = 2,260 gm, *m_2_* = 720 gm, *m_3_* = 680 gm, *m_4_* = 640 gm, and *m_5_* = 600 gm. Figure 9(a) shows the mass of each link with its motor for the manipulator of the proposed design.

For the conventional three dimensional planar manipulator (one motor for each link), the mass of the first link will equal to the mass of link itself plus the mass of the motor which controls the second link position, *i.e.*, 760 + 1,500 gm. The mass of the second link will equal to the mass of link itself plus the mass of the motor which controls the third link position, *i.e.*, 720 + 1,500 gm. The mass of the third link will equal to the mass of third link plus the mass of the motor which controls the fourth link position, *i.e.*, 680 + 1,500 gm. The mass of the fourth link will equal to the mass of fourth link plus the mass of the motor which controls the fifth link position, *i.e.*, 640 + 1,500 gm, while the mass of the last link will equal to the mass of the link itself because there are no more motors, *i.e.*, 600 gm. Figure 9(b) shows the mass of each link using the manipulator with five motors, while Table 2 shows the values of mass of the links using both the manipulator with two motors and the manipulator with five links.

It is clearly noted how the proposed method could be used to decrease the weight of manipulator. Decreasing the weight leads to a decrease of the torques of each link. The next section shows the results of the torques of each joint when the end-effector is following a desired path. Using the Lagrangian formulation, the dynamical equations of motion of the manipulator is:

(10) $$\sum_{j=1}^{6}M_{ij}{\ddot{q}}_{j}+V_{i}+G_{i}=Q_{i}$$

for *i* = 1,2,….,6.

The first term in this equation is the inertia forces, the second term represents the Coriolis and centrifugal forces, and the third term gives the gravitational effects [1,20,21]. Dynamics equations of the manipulator are discussed in details in the Appendix.

As shown by dynamics equations, increasing the weight of motors will increase the torques needed to control the manipulator. In order to decrease the effect of the motors weight on the inertia of manipulators, parallel manipulators are used, as we mentioned earlier. For example in reference [22], the parallel manipulator is actuated by three servo-motors located at the base which contributes to reducing the inertia of manipulators. Reference [23] shows another way to decrease the effect of the motors weight on the inertia of manipulators. This reference shows a simple configuration design, which comprises of only three joints: two at the shoulder and one at the hand. In this design, the moment of inertia of the arm is constant and independent from the joint angles. In contrast for our manipulator, we see from Equations A21–A25 that the moment of inertia value is dependent on the joint angles.

## 4. Simulation Results

This section shows the effectiveness of using the proposed manipulator to be used when it is desired to make the end-effector follow a desired path. This section has two examples. The first example calculates the torques using both manipulators (the proposed one and the conventional three dimensional planar manipulator) and shows how effective the proposed manipulator is in decreasing the torque of each joint required to move the manipulator. To verify the estimation results and compare between them and the results measured from the manipulator itself, the second example has been shown. This example shows the results if the torque using: (1) the conventional three dimensional planar manipulator with defined desired joint angles path, (2) the proposed manipulator with the defined desired joint angles path and finally (3) the proposed manipulator with the measured joint angles path when the joint angles follow the desired joint angles path.

### Case One

Torque of each joint for both the manipulators is calculated to show the effectiveness of using the proposed manipulator to decrease the torque of each joint. Using the same manipulator with *l* = [19,18,17,16,15]*^T^*, and *d_2_* = 21 where all lengths are in cm, the joint angles path is defined as:

(11) $$\theta_{1}(t)=-0.5\cos(4t)$$

(12) $$\theta_{2}(t)=-\cos(2t)+1$$

(13) $$\theta_{3}(t)=-4\cos(t)+3$$

**Figure 10.**
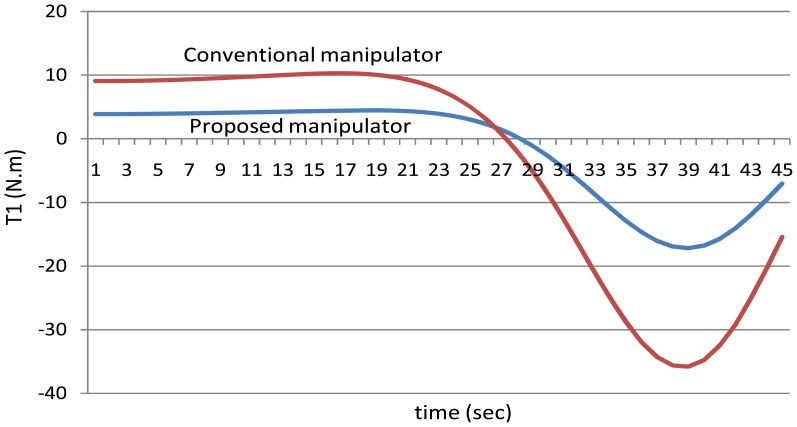
The values of the torques of the first joint using the both manipulators.

It should be remembered that when using the proposed manipulator, *θ_3_*, *θ_4_*, *θ_5_*, *θ_6_* are equal. To show the effectiveness of the proposed manipulator in decreasing the torque, Figure 10 shows the values of the torques of the first joint using both the manipulators, the proposed manipulator (with three motors) and the manipulator of six motors.

**Figure 11.**
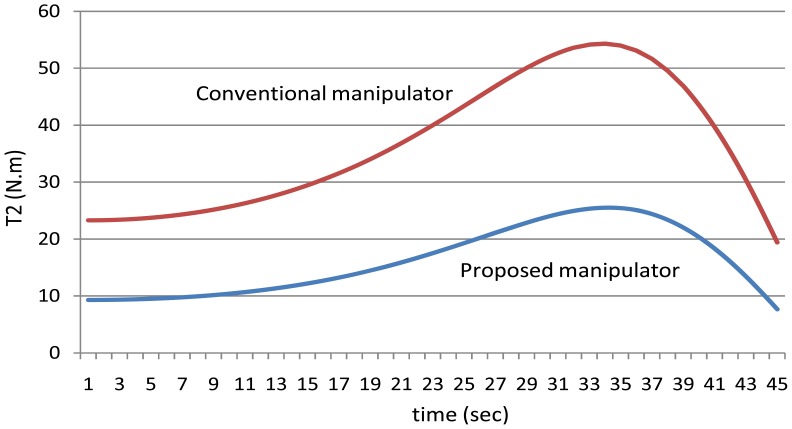
The values of the torques of the second joint using the both manipulators.

Figure 11 shows the values of the torques of the second joint using the manipulators.

**Figure 12.**
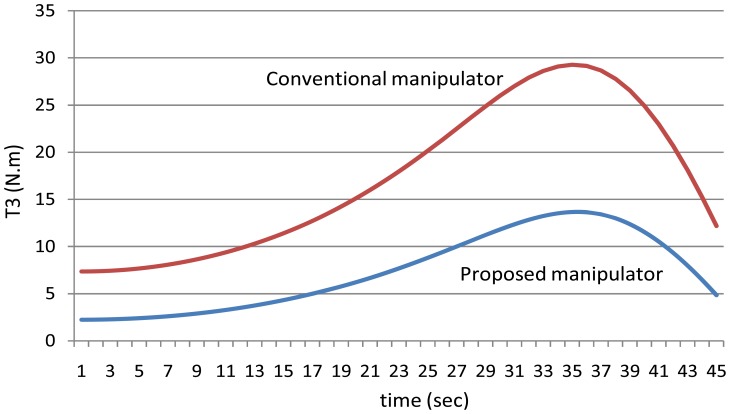
The values of the torques of the third joint using the both manipulators.

**Figure 13.**
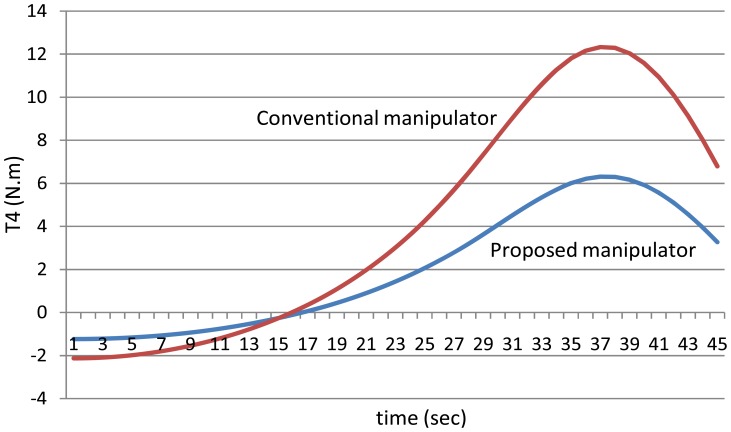
The values of the torques of the fourth joint using the both manipulators.

Figure 12 shows the absolute values of the torques of the third joint while Figure 13 shows the absolute values of the torques of the fourth joint using the both manipulators.

**Figure 14.**
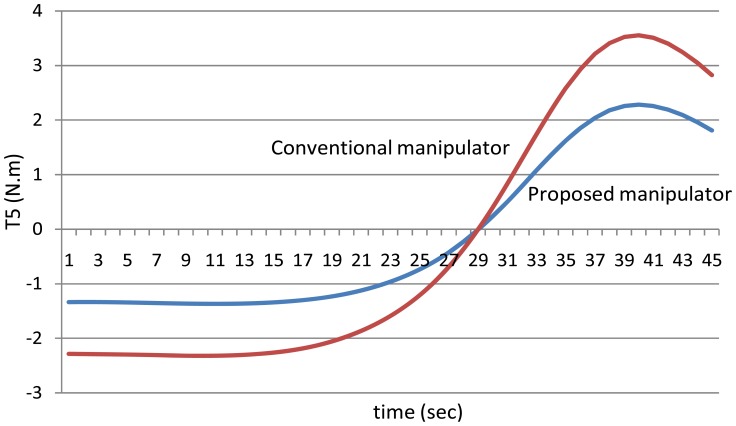
The values of the torques of the fifth joint using the both manipulators.

**Figure 15.**
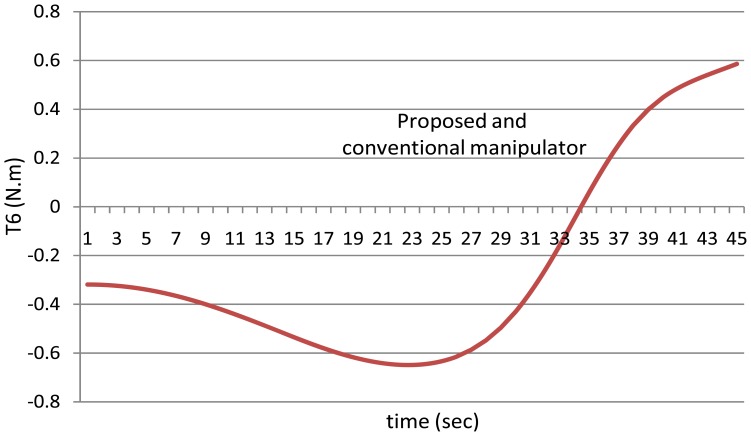
The values of the torques of the sixth joint using the both manipulators.

Figure 14 shows the torques of the fifth joint and finally Figure 15 shows the torques of the sixth joint angle using the both manipulators.

First of all, it is noted that the torque of the sixth joint has the same value using both the manipulators because the sixth link has the same mass for both the manipulators, in other words the mass of the sixth link is equal to the mass of the link itself only because it does not hold any motor.

**Figure 16.**
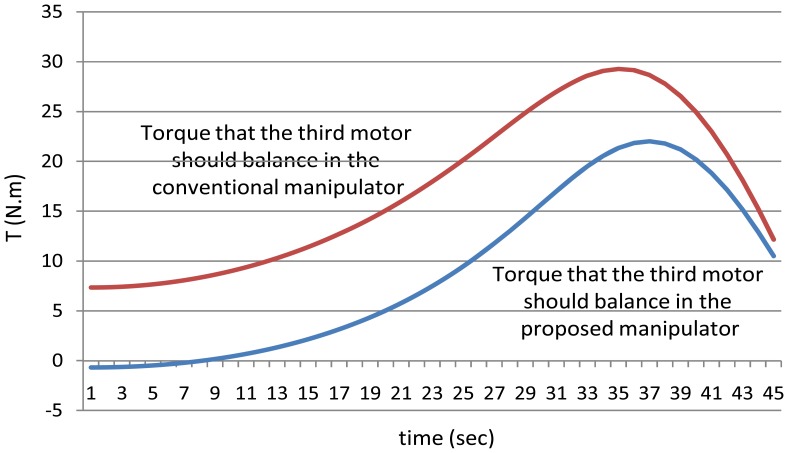
The values of the torques of the third motor using the both manipulators.

Secondly, as mentioned earlier for the proposed manipulator, the third motor should balance the torque of all the third, fourth, fifth and the sixth joint. In other words, the torque of the third motor should equal to (T_3_ + T_4_ + T_5_ + T_6_) for the proposed manipulator. Figure 16 shows the power that the third motor should balance for both the manipulators. It is noted from this example that using the proposed manipulator not only decreases the number of motors used in the manipulator, but also decreases the torques of the motors used to control it.

### Case Two

The trajectory applied to robot in verification experiments in this case is:

(13) $$\theta_{1}(t)=\frac{e^{0.7t}}{25}$$

(14) $$\theta_{2}(t)=\frac{e^{0.5t}}{3}$$

(15) $$\theta_{3}(t)=\frac{e^{0.9t}}{150}$$

**Figure 17.**
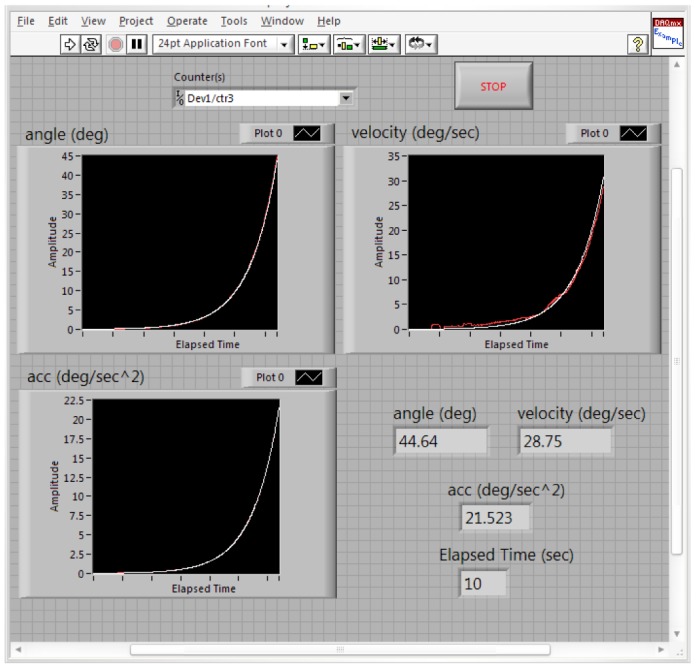
The values of the estimated and measured angular position, velocity and acceleration of the first joint angle (white: estimated, red: measured).

**Figure 18.**
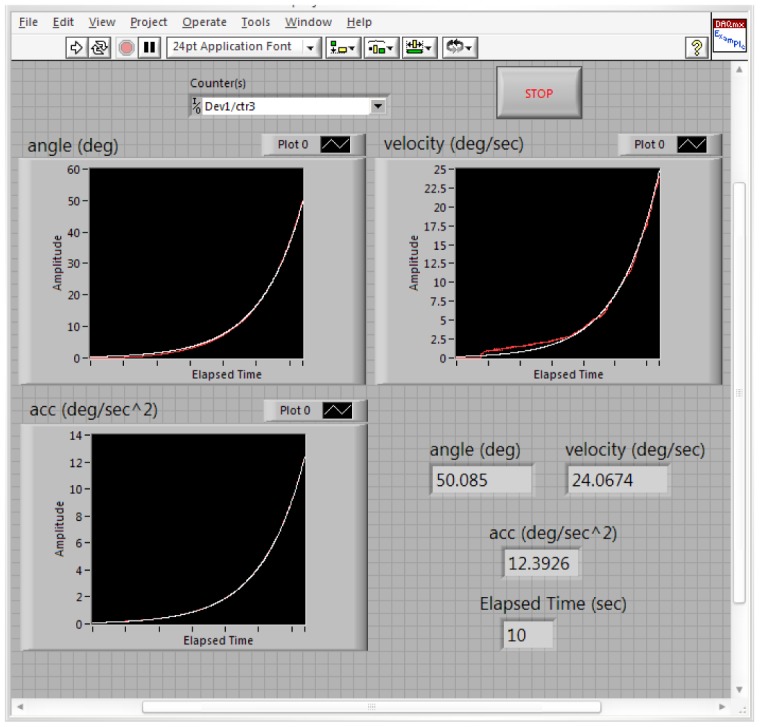
The values of the estimated and measured angular position, velocity and acceleration of the second joint angle (white: estimated, red: measured).

Figure 17 shows the estimated (white) and measured (red) angle, angular velocity and angular acceleration of the first joint angle defined above. Figure 18 shows the estimated (white) and measured (red) angle, angular velocity and angular acceleration of the second joint angle.

**Figure 19.**
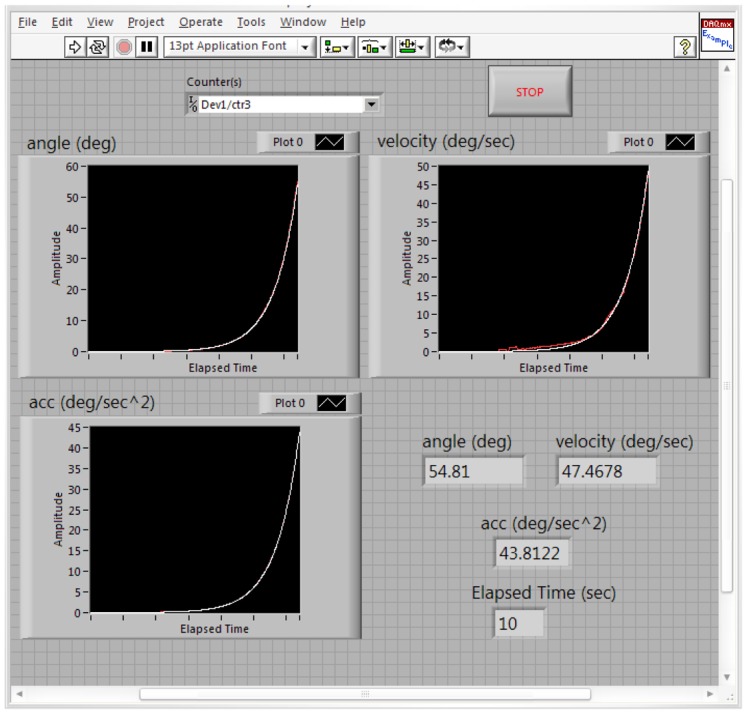
The values of the estimated and measured angular position, velocity and acceleration of the third joint angle (white: estimated, red: measured).

Figure 19 shows the estimated (white) and measured (red) angle, angular velocity and angular acceleration of the third joint angle of the manipulator. It should be remembered again that using the proposed manipulator, *θ_3_*, *θ_4_*, *θ_5_*, *θ_6_* are equals.

**Figure 20.**
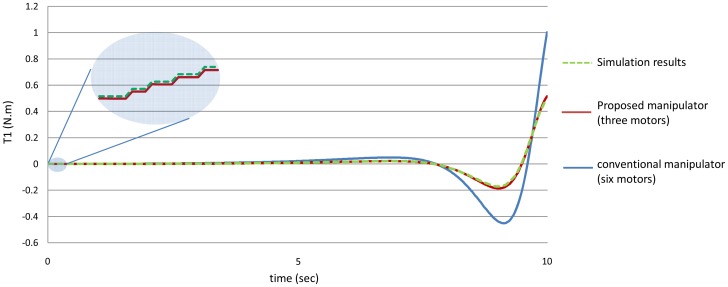
The torque of the first joint angle.

**Figure 21.**
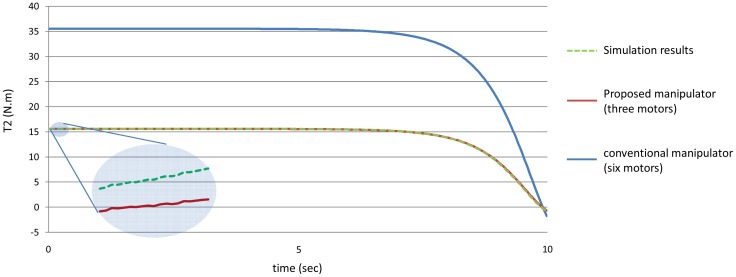
The torque of the second joint angle.

**Figure 22.**
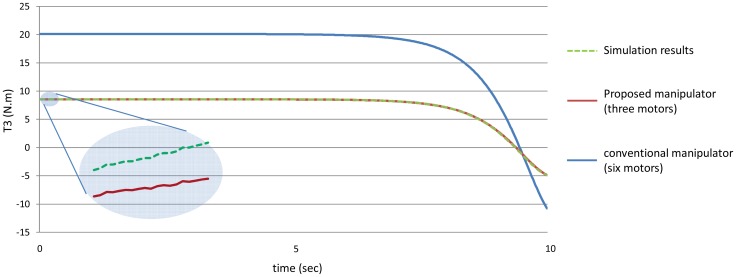
The torque of the third joint angle.

**Figure 23.**
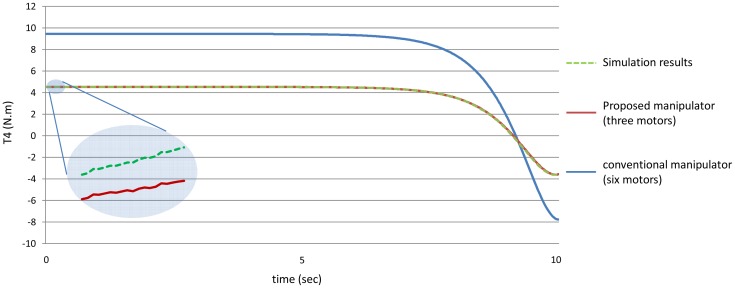
The torque of the fourth joint angle.

**Figure 24.**
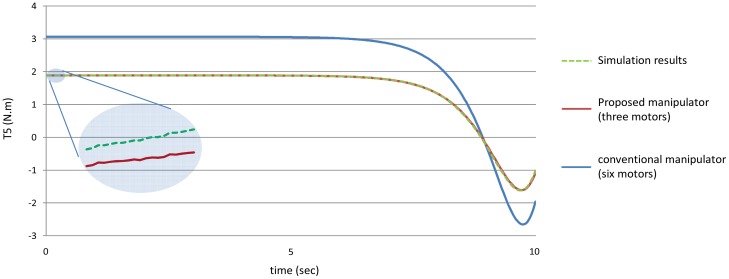
The torque of the fifth joint angle.

**Figure 25.**
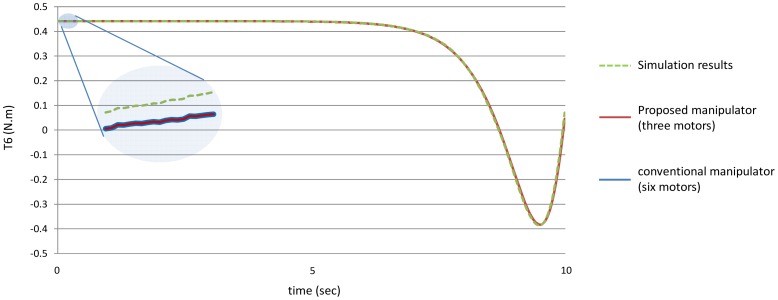
The torque of the sixth joint angle.

Figures 20–25 show the comparison between the torque of each joint angle for: (1) the conventional three dimensional planar manipulator using the estimated joint angles path; (2) the proposed manipulator using the estimated joint angles path; and finally (3) the proposed manipulator using the measured angular position, velocity and acceleration of the manipulator joints.

The results obtained from verification experiments indicate that there is a good agreement between the torque of the joint angles for the proposed manipulator using the estimated joint angles path (green) and the measured joint angles path (red). These figures show the effectiveness of the proposed manipulator in decreasing the torque of the joint angles using the proposed manipulator.

**Figure 26.**
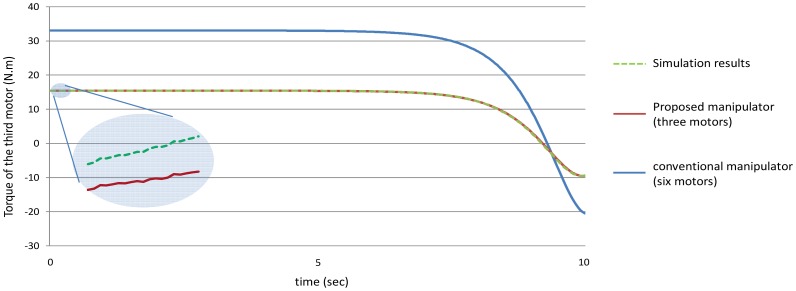
The torque of the third motor.

As mentioned in the first example that for the proposed manipulator, the third motor should balance the torque of all the third, fourth, fifth and the sixth joint, *i.e.*, the torque of the third motor should equal (T_3_ + T_4_ + T_5_ + T_6_) for the proposed manipulator, Figure 26 shows that even though that this motor (third motor) should balance the torques of four links, this motor could be smaller in size (less power) in the proposed manipulator than the third motor in the conventional three dimensional planar manipulator.

## 5. Conclusions

This paper presents a mechanical design for a three dimensional planar redundant manipulator. Theoretically, for each degree of freedom there should be one motor. However, in this design only three motors are needed to control any *n* degrees of freedom three dimensional planar redundant manipulator. Therefore, this design can be used to decrease the weight of the manipulator significantly. The design steps of this manipulator are explained in detail. The dynamical equations are calculated for both the proposed and the conventional three dimensional planar manipulators (with *n* motors) and it is concluded from the result, that even though the proposed manipulator has less motors, these motors could be even smaller (as regard to power) than the motors used with conventional three dimensional planar manipulators.

## Figures and Tables

**Table 1.** Link parameters of the manipulator.

i	α	a	d	θ
1	90	0	0	*θ1*
2	0	*l1*	*d1*	*θ2*
3	0	*l2*	0	*θ3*
4	0	*l3*	0	*θ4*
5	0	*l4*	0	*θ5*
6	0	*l5*	0	*θ6*

**Table 2.** The mass of links for both the proposed and conventional manipulators.

**m1(gm)**	**1,500**	**1,500**

m2(gm)	2,260	2,260
m3(gm)	720	2,220
m4(gm)	680	2,180
m5(gm)	640	2,140
m6(gm)	600	600

## Appendix

This section explains the Dynamics of the manipulator used in experiments. In Equation (10):

(A1) $$M=\sum_{i=1}^{6}\left(J_{\upsilon i}^{T}m_{i}J_{\upsilon i}+J_{wi}^{T}I_{i}J_{wi}\right)$$

where *J_υi_* is the Jacobian submatrix assosiated with the linear velocity of the center of mass of link *i* and *J_wi_* is the Jacobian submatrix assosiated with the angular velocity vector of link *i*.

(A2) $$J_{\upsilon i}=\left[J_{\upsilon i}^{1},J_{\upsilon i}^{2},\ldots ,J_{\upsilon i}^{i},0,0,\ldots ,0\right]$$

(A3) $$J_{wi}=\left[J_{wi}^{1},J_{wi}^{2},\ldots ,J_{wi}^{i},0,0,\ldots ,0\right]$$

where $J_{\upsilon i}^{j}$ and $J_{wi}^{j}$ are the *j* th column vectors of *J_υi_* and *J_wi_*, respectively. Applying the theory of instantaneous screw motion [1] for *j* ≤ *i*, and because all the joints are revolute joints, we obtain:

(A4) $$J_{\upsilon i}^{j}=z_{j-1}\times^{j-1}p_{ci}^{*}$$

(A5) $$J_{wi}^{j}=z_{j-1}$$

The link Jacobian submatrices, *J_υi_* for the proposed manipulator can be obtained as:

(A6) $$J_{\upsilon 1}=\left[\begin{array}{cccccc} 0 & 0 & 0 & 0 & 0 & 0\\ 0 & 0 & 0 & 0 & 0 & 0\\ 0 & 0 & 0 & 0 & 0 & 0 \end{array}\right]$$

*J_υ1_* is a zero matrix because from the link parameters of the manipulator of Table 1, it is noted that *a_1_* and *d_1_* are equal to zero because the first motor is set on the base.

(A7) $$J_{\upsilon 2}=\left[\begin{array}{cccccc} d_{1}c_{1}-\frac{l_{1}}{2}s_{1}c_{2} & -\frac{l_{1}}{2}c_{1}s_{2} & 0 & 0 & 0 & 0\\ d_{1}s_{1}-\frac{l_{1}}{2}c_{1}c_{2} & -\frac{l_{1}}{2}s_{1}s_{2} & 0 & 0 & 0 & 0\\ 0 & \frac{l_{1}}{2}c_{2} & 0 & 0 & 0 & 0 \end{array}\right]$$

(A8) $$J_{\upsilon 3}=\left[\begin{array}{cccccc} d_{1}c_{1}-s_{1}\left(l_{1}c_{2}+\frac{l_{2}}{2}c_{23}\right) & -c_{1}\left(l_{1}s_{2}+\frac{l_{2}}{2}s_{23}\right) & -c_{1}\left(\frac{l_{2}}{2}s_{23}\right) & 0 & 0 & 0\\ d_{1}s_{1}+c_{1}\left(l_{1}c_{2}+\frac{l_{2}}{2}c_{23}\right) & -s_{1}\left(l_{1}s_{2}+\frac{l_{2}}{2}s_{23}\right) & -s_{1}\left(\frac{l_{2}}{2}s_{23}\right) & 0 & 0 & 0\\ 0 & l_{1}c_{2}+\frac{l_{2}}{2}c_{23} & \frac{l_{2}}{2}c_{23} & 0 & 0 & 0 \end{array}\right]$$

(A9) $$J_{\upsilon 4}=\left[\begin{array}{cccccc} d_{1}c_{1}-s_{1}\left(l_{1}c_{2}+l_{2}c_{23}+\frac{l_{3}}{2}c_{234}\right) & -c_{1}\left(l_{1}s_{2}+l_{2}s_{23}+\frac{l_{3}}{2}s_{234}\right) & -c_{1}\left(l_{2}s_{23}+\frac{l_{3}}{2}s_{234}\right) & -c_{1}\left(\frac{l_{3}}{2}s_{234}\right) & 0 & 0\\ d_{1}s_{1}+c_{1}\left(l_{1}c_{2}+l_{2}c_{23}+\frac{l_{3}}{2}c_{234}\right) & -s_{1}\left(l_{1}s_{2}+l_{2}s_{23}+\frac{l_{3}}{2}s_{234}\right) & -s_{1}\left(l_{2}s_{23}+\frac{l_{3}}{2}s_{234}\right) & -s_{1}\left(\frac{l_{3}}{2}s_{234}\right) & 0 & 0\\ 0 & l_{1}c_{2}+l_{2}c_{23}+\frac{l_{3}}{2}c_{234} & l_{2}c_{23}+\frac{l_{3}}{2}c_{234} & \frac{l_{3}}{2}c_{234} & 0 & 0 \end{array}\right]$$

(A10) $$J_{\upsilon 5}=\left[\begin{array}{cccccc} d_{1}c_{1}-s_{1}\left(l_{1}c_{2}+l_{2}c_{23}+l_{3}c_{234}+\frac{l_{4}}{2}c_{2345}\right) & -c_{1}\left(l_{1}s_{2}+l_{2}s_{23}+l_{3}s_{234}+\frac{l_{4}}{2}s_{2345}\right) & -c_{1}\left(l_{2}s_{23}+l_{3}s_{234}+\frac{l_{4}}{2}s_{2345}\right) & -c_{1}\left(l_{3}s_{234}+\frac{l_{4}}{2}s_{2345}\right) & -c_{1}\left(\frac{l_{4}}{2}s_{2345}\right) & 0\\ d_{1}s_{1}+c_{1}\left(l_{1}c_{2}+l_{2}c_{23}+l_{3}c_{234}+\frac{l_{4}}{2}c_{2345}\right) & -s_{1}\left(l_{1}s_{2}+l_{2}s_{23}+l_{3}s_{234}+\frac{l_{4}}{2}s_{2345}\right) & -s_{1}\left(l_{2}s_{23}+l_{3}s_{234}+\frac{l_{4}}{2}s_{2345}\right) & -s_{1}\left(l_{3}s_{234}+\frac{l_{4}}{2}s_{2345}\right) & -s_{1}\left(\frac{l_{4}}{2}s_{2345}\right) & 0\\ 0 & l_{1}c_{2}+l_{2}c_{23}+l_{3}c_{234}+\frac{l_{4}}{2}c_{2345} & l_{2}c_{23}+l_{3}c_{234}+\frac{l_{4}}{2}c_{2345} & l_{3}c_{234}+\frac{l_{4}}{2}c_{2345} & \frac{l_{4}}{2}c_{2345} & 0 \end{array}\right]$$

(A11) $$J_{\upsilon 6}=\left[\begin{array}{cccccc} d_{1}c_{1}-s_{1}\left(l_{1}c_{2}+l_{2}c_{23}+l_{3}c_{234}+l_{4}c_{2345}+\frac{l_{5}}{2}c_{23456}\right) & -c_{1}\left(l_{1}s_{2}+l_{2}s_{23}+l_{3}s_{234}+l_{4}s_{2345}+\frac{l_{5}}{2}s_{23456}\right) & -c_{1}\left(l_{2}s_{23}+l_{3}s_{234}+l_{4}s_{2345}+\frac{l_{5}}{2}s_{23456}\right) & -c_{1}\left(l_{3}s_{234}+l_{4}s_{2345}+\frac{l_{5}}{2}s_{23456}\right) & -c_{1}\left(l_{4}s_{2345}+\frac{l_{5}}{2}s_{23456}\right) & -c_{1}\left(\frac{l_{5}}{2}s_{23456}\right)\\ d_{1}s_{1}+c_{1}\left(l_{1}c_{2}+l_{2}c_{23}+l_{3}c_{234}+l_{4}c_{2345}+\frac{l_{5}}{2}c_{23456}\right) & -s_{1}\left(l_{1}s_{2}+l_{2}s_{23}+l_{3}s_{234}+l_{4}s_{2345}+\frac{l_{5}}{2}s_{23456}\right) & -s_{1}\left(l_{2}s_{23}+l_{3}s_{234}+l_{4}s_{2345}+\frac{l_{5}}{2}s_{23456}\right) & -s_{1}\left(l_{3}s_{234}+l_{4}s_{2345}+\frac{l_{5}}{2}s_{23456}\right) & -s_{1}\left(l_{4}s_{2345}+\frac{l_{5}}{2}s_{23456}\right) & -s_{1}\left(\frac{l_{5}}{2}s_{23456}\right)\\ 0 & l_{1}c_{2}+l_{2}c_{23}+l_{3}c_{234}+l_{4}c_{2345}+\frac{l_{5}}{2}c_{23456} & l_{2}c_{23}+l_{3}c_{234}+l_{4}c_{2345}+\frac{l_{5}}{2}c_{23456} & l_{3}c_{234}+l_{4}c_{2345}+\frac{l_{5}}{2}c_{23456} & l_{4}c_{2345}+\frac{l_{5}}{2}c_{23456} & \frac{l_{5}}{2}c_{23456} \end{array}\right]$$

To formulate the submatrices of *J_wi_*, following equations are used:

(A12) $$J_{w1}=\left[\begin{array}{cccccc} 0 & 0 & 0 & 0 & 0 & 0\\ 0 & 0 & 0 & 0 & 0 & 0\\ 1 & 0 & 0 & 0 & 0 & 0 \end{array}\right]$$

(A13) $$J_{w2}=\left[\begin{array}{cccccc} 0 & s_{1} & 0 & 0 & 0 & 0\\ 0 & -c_{1} & 0 & 0 & 0 & 0\\ 1 & 0 & 0 & 0 & 0 & 0 \end{array}\right]$$

(A44) $$J_{w3}=\left[\begin{array}{cccccc} 0 & s_{1} & s_{1} & 0 & 0 & 0\\ 0 & -c_{1} & -c_{1} & 0 & 0 & 0\\ 1 & 0 & 0 & 0 & 0 & 0 \end{array}\right]$$

(A15) $$J_{w4}=\left[\begin{array}{cccccc} 0 & s_{1} & s_{1} & s_{1} & 0 & 0\\ 0 & -c_{1} & -c_{1} & -c_{1} & 0 & 0\\ 1 & 0 & 0 & 0 & 0 & 0 \end{array}\right]$$

(A16) $$J_{w5}=\left[\begin{array}{cccccc} 0 & s_{1} & s_{1} & s_{1} & s_{1} & 0\\ 0 & -c_{1} & -c_{1} & -c_{1} & -c_{1} & 0\\ 1 & 0 & 0 & 0 & 0 & 0 \end{array}\right]$$

(A17) $$J_{w6}=\left[\begin{array}{cccccc} 0 & s_{1} & s_{1} & s_{1} & s_{1} & s_{1}\\ 0 & -c_{1} & -c_{1} & -c_{1} & -c_{1} & -c_{1}\\ 1 & 0 & 0 & 0 & 0 & 0 \end{array}\right]$$

Now to formulate the inertia matrices of the manipulator, assuming that the moving links are homogeneous with relatively small cross section, the inertia matrix *^i^I_i_* of link *i* about its center of mass and expressed in the link link frame *i* th link fram is:

(A18) Iii=112miai2[000010001]

for *I* = 1,2,…,6.

Now to find Ii inertia matrix of link i about its center of mass and expressed in the base link frame, the following equation is used:

(A19) Ii=R0iIii(R0i)T

Using Equations (A18) and (A19), we obtain:

(A20) $$I_{1}=\left[\begin{array}{ccc} 0 & 0 & 0\\ 0 & 0 & 0\\ 0 & 0 & 0 \end{array}\right]$$

*I_1_* is a zero matrix because *a_1_* is equal to zero.

(A21) $$I_{2}=\frac{m_{2}l_{1}^{2}}{12}\left[\begin{array}{ccc} s_{1}^{2}+c_{1}^{2}c_{2}^{2} & s_{1}c_{1}\left(s_{2}^{2}-1\right) & -c_{1}\left(s_{2}c_{2}\right)\\ s_{1}c_{1}\left(s_{2}^{2}-1\right) & c_{1}^{2}+s_{1}^{2}s_{2}^{2} & -s_{1}\left(s_{2}c_{2}\right)\\ -c_{1}\left(s_{2}c_{2}\right) & -s_{1}\left(s_{2}c_{2}\right) & c_{2}^{2} \end{array}\right]$$

(A22) $$I_{3}=\frac{m_{3}l_{2}^{2}}{12}\left[\begin{array}{ccc} s_{1}^{2}+c_{1}^{2}c_{23}^{2} & s_{1}c_{1}\left(s_{23}^{2}-1\right) & -c_{1}\left(s_{23}c_{23}\right)\\ s_{1}c_{1}\left(s_{23}^{2}-1\right) & c_{1}^{2}+s_{1}^{2}s_{23}^{2} & -s_{1}\left(s_{23}c_{23}\right)\\ -c_{1}\left(s_{23}c_{23}\right) & -s_{1}\left(s_{23}c_{23}\right) & c_{23}^{2} \end{array}\right]$$

(A23) $$I_{4}=\frac{m_{4}l_{3}^{2}}{12}\left[\begin{array}{ccc} s_{1}^{2}+c_{1}^{2}c_{234}^{2} & s_{1}c_{1}\left(s_{234}^{2}-1\right) & -c_{1}\left(s_{234}c_{234}\right)\\ s_{1}c_{1}\left(s_{234}^{2}-1\right) & c_{1}^{2}+s_{1}^{2}s_{234}^{2} & -s_{1}\left(s_{234}c_{234}\right)\\ -c_{1}\left(s_{234}c_{234}\right) & -s_{1}\left(s_{234}c_{234}\right) & c_{234}^{2} \end{array}\right]$$

(A24) $$I_{5}=\frac{m_{5}l_{4}^{2}}{12}\left[\begin{array}{ccc} s_{1}^{2}+c_{1}^{2}c_{2345}^{2} & s_{1}c_{1}\left(s_{2345}^{2}-1\right) & -c_{1}\left(s_{2345}c_{2345}\right)\\ s_{1}c_{1}\left(s_{2345}^{2}-1\right) & c_{1}^{2}+s_{1}^{2}s_{2345}^{2} & -s_{1}\left(s_{2345}c_{2345}\right)\\ -c_{1}\left(s_{2345}c_{2345}\right) & -s_{1}\left(s_{2345}c_{2345}\right) & c_{2345}^{2} \end{array}\right]$$

(A25) $$I_{6}=\frac{m_{6}l_{5}^{2}}{12}\left[\begin{array}{ccc} s_{1}^{2}+c_{1}^{2}c_{23456}^{2} & s_{1}c_{1}\left(s_{23456}^{2}-1\right) & -c_{1}\left(s_{23456}c_{23456}\right)\\ s_{1}c_{1}\left(s_{23456}^{2}-1\right) & c_{1}^{2}+s_{1}^{2}s_{23456}^{2} & -s_{1}\left(s_{23456}c_{23456}\right)\\ -c_{1}\left(s_{23456}c_{23456}\right) & -s_{1}\left(s_{23456}c_{23456}\right) & c_{23456}^{2} \end{array}\right]$$

Now to find the inertia manipulator matrix *M*, Equation (A1) can be rewritten as:

(A26) $$\begin{array}{l} M=J_{\upsilon 1}^{T}m_{1}J_{\upsilon 1}+J_{w1}^{T}I_{1}J_{w1}+J_{\upsilon 2}^{T}m_{2}J_{\upsilon 2}+J_{w2}^{T}I_{2}J_{w2}+J_{\upsilon 3}^{T}m_{3}J_{\upsilon 3}+J_{w3}^{T}I_{3}J_{w3}+J_{\upsilon 4}^{T}m_{4}J_{\upsilon 4}+J_{w4}^{T}I_{4}J_{w4}\\ +J_{\upsilon 5}^{T}m_{5}J_{\upsilon 5}+J_{w5}^{T}I_{5}J_{w5}+J_{\upsilon 6}^{T}m_{6}J_{\upsilon 6}+J_{w6}^{T}I_{6}J_{w6} \end{array}$$

Therefore, Equation (10) can be rewritten as:

(A27) $$\left[\begin{array}{c} \tau_{1}\\ \tau_{2}\\ \tau_{3}\\ \tau_{4}\\ \tau_{5}\\ \tau_{6} \end{array}\right]=\left[\begin{array}{cccccc} M_{11} & M_{12} & M_{13} & M_{14} & M_{15} & M_{16}\\ M_{21} & M_{22} & M_{23} & M_{24} & M_{25} & M_{26}\\ M_{31} & M_{32} & M_{33} & M_{34} & M_{35} & M_{36}\\ M_{41} & M_{42} & M_{43} & M_{44} & M_{45} & M_{46}\\ M_{51} & M_{52} & M_{53} & M_{54} & M_{55} & M_{56}\\ M_{61} & M_{62} & M_{63} & M_{64} & M_{65} & M_{66} \end{array}\right]\left[\begin{array}{c} {\ddot{\theta }}_{1}\\ {\ddot{\theta }}_{2}\\ {\ddot{\theta }}_{3}\\ {\ddot{\theta }}_{4}\\ {\ddot{\theta }}_{5}\\ {\ddot{\theta }}_{6} \end{array}\right]+\left[\begin{array}{c} V_{1}(\theta ,\ddot{\theta })\\ V_{2}(\theta ,\ddot{\theta })\\ V_{3}(\theta ,\ddot{\theta })\\ V_{4}(\theta ,\ddot{\theta })\\ V_{5}(\theta ,\ddot{\theta })\\ V_{6}(\theta ,\ddot{\theta }) \end{array}\right]+\left[\begin{array}{c} G_{1}(\theta )\\ G_{2}(\theta )\\ G_{3}(\theta )\\ G_{4}(\theta )\\ G_{5}(\theta )\\ G_{6}(\theta ) \end{array}\right]$$

where:

$$\begin{array}{ll} {\text{M}}_{11}= & \frac{1}{12}(4m_{2}(3d_{1}^{2}+l_{1}^{2}\cos^{2}(\theta_{2}))+4m_{3}(3d_{1}^{2}+3l_{1}^{2}\cos^{2}(\theta_{2})+l_{2}^{2}\cos^{2}(\theta_{2}+\theta_{3})+3l_{1}l_{2}\cos(\theta_{2})\cos(\theta_{2}+\theta_{3}))\\ & +12m_{4}(d_{1}^{2}+\frac{1}{4}{\left(2l_{1}\cos(\theta_{2})+2l_{2}\cos(\theta_{2}+\theta_{3})+l_{3}\cos(\theta_{2}+\theta_{3}+\theta_{4})\right)}^{2}+12m_{s}(d_{1}^{2}\\ & +\frac{1}{4}{\left(2l_{1}\cos(\theta_{2})+2l_{2}\cos(\theta_{2}+\theta_{3})+2l_{3}\cos(\theta_{2}+\theta_{3}+\theta_{4})+l_{4}\cos(\theta_{2}+\theta_{3}+\theta_{4}+\theta_{5})\right)}^{2})\\ & +12m_{6}(d_{1}^{2}\\ & +\frac{1}{4}(2(l_{1}\cos(\theta_{2})+l_{2}\cos(\theta_{2}+\theta_{3})+l_{3}\cos(\theta_{2}+\theta_{3}+\theta_{4})+l_{4}\cos(\theta_{2}+\theta_{3}+\theta_{4}+\theta_{5}))+l_{5}\cos(\theta_{2}\\ & {+\theta_{3}+\theta_{4}+\theta_{5}+\theta_{6}))}^{2})+l_{3}^{2}m_{4}\cos^{2}(\theta_{2}+\theta_{3}+\theta_{4})+l_{4}^{2}m_{5}\cos^{2}(\theta_{2}+\theta_{3}+\theta_{4}+\theta_{5})+l_{5}^{2}m_{6}\cos^{2}(\theta_{2}\\ & +\theta_{3}+\theta_{4}+\theta_{5}+\theta_{6})) \end{array}$$

$$\begin{array}{ll} {\text{M}}_{12}={\text{M}}_{21}= & \frac{1}{2}d_{1}(l_{3}m_{4}\sin(\theta_{2}+\theta_{3}+\theta_{4})+2l_{3}m_{5}\sin(\theta_{2}+\theta_{3}+\theta_{4})+l_{4}m_{5}\sin(\theta_{2}+\theta_{3}+\theta_{4}+\theta_{5})\\ & +m_{6}(2l_{3}\sin(\theta_{2}+\theta_{3}+\theta_{4})+2l_{4}\sin(\theta_{2}+\theta_{3}+\theta_{4}+\theta_{5})+l_{5}\sin(\theta_{2}+\theta_{3}+\theta_{4}+\theta_{5}+\theta_{6}))\\ & +l_{2}(m_{2}+2(m_{4}+m_{5}+m_{6}))\sin(\theta_{2}+\theta_{3})+l_{1}(m_{3}+2(m_{3}+m_{4}+m_{5}+m_{6}))\sin(\theta_{2})) \end{array}$$

$$\begin{array}{ll} {\text{M}}_{13}={\text{M}}_{31}= & -\frac{1}{2}d_{1}(l_{4}m_{5}\sin(\theta_{2}+\theta_{3}+\theta_{4}+\theta_{5})+m_{6}(2l_{4}\sin(\theta_{2}+\theta_{3}+\theta_{4}+\theta_{5})+l_{5}\sin(\theta_{2}+\theta_{3}+\theta_{4}\\ & +\theta_{5}+\theta_{6}))+l_{3}(m_{4}+2(m_{5}+m_{6}))\sin(\theta_{2}+\theta_{3}+\theta_{4})+l_{2}(m_{3}+2(m_{4}+m_{5}\\ & +m_{6}))\sin(\theta_{2}+\theta_{3})) \end{array}$$

$$\begin{array}{ll} {\text{M}}_{14}={\text{M}}_{41}= & -\frac{1}{2}d_{1}(l_{5}m_{6}\sin(\theta_{2}+\theta_{3}+\theta_{4}+\theta_{5}+\theta_{6})+l_{4}(m_{5}+2m_{6})\sin(\theta_{2}+\theta_{3}+\theta_{4}+\theta_{5})+l_{3}(m_{4}\\ & +2(m_{5}+m_{6}))\sin(\theta_{2}+\theta_{3}+\theta_{4})) \end{array}$$

$${\text{M}}_{15}={\text{M}}_{51}=-\frac{1}{2}{\text{d}}_{1}(l_{5}{\text{m}}_{6}\sin(\theta_{2}+\theta_{3}+\theta_{4}+\theta_{5}+\theta_{6})+l_{4}({\text{m}}_{5}+2{\text{m}}_{6})\sin(\theta_{2}+\theta_{3}+\theta_{4}+\theta_{5}))$$

$${\text{M}}_{16}=-\frac{1}{2}d_{1}l_{5}m_{6}\sin(\theta_{2}+\theta_{3}+\theta_{4}+\theta_{5}+\theta_{6})$$

$$\begin{array}{ll} {\text{M}}_{22}= & \frac{1}{192}(192l_{1}(l_{4}m_{5}\cos(\theta_{3}+\theta_{4}+\theta_{5})+m_{6}(2l_{4}\cos(\theta_{3}+\theta_{4}+\theta_{5})+l_{5}\cos(\theta_{3}+\theta_{4}+\theta_{5}+\theta_{6}))\\ & +l_{3}(m_{4}+2(m_{5}+m_{6}))\cos(\theta_{3}+\theta_{4})+l_{2}(m_{3}+2(m_{4}+m_{5}+m_{6}))\cos(\theta_{3}))\\ & +2l_{3}^{2}m_{4}\cos(2(\theta_{2}+\theta_{3}+\theta_{4}))-l_{3}^{2}m_{4}\cos(2(2\theta_{1}+\theta_{2}+\theta_{3}+\theta_{4}))-l_{3}^{2}m_{4}\cos(4\theta_{1}-2(\theta_{2}\\ & +\theta_{3}+\theta_{4}))+2l_{4}^{2}m_{5}\cos(2(\theta_{2}+\theta_{3}+\theta_{4}+\theta_{5}))-l_{4}^{2}m_{5}\cos(2(2\theta_{1}+\theta_{2}+\theta_{3}+\theta_{4}+\theta_{5}))\\ & -l_{4}^{2}m_{5}\cos(4\theta_{1}-2(\theta_{2}+\theta_{3}+\theta_{4}+\theta_{5}))+192l_{3}l_{4}m_{5}\cos(\theta_{5})+2m_{6}(l_{5}^{2}(32-(\cos(4\theta_{1})\\ & -1)\cos(2(\theta_{2}+\theta_{3}+\theta_{4}+\theta_{5}+\theta_{6})))+96l_{5}(l_{3}\cos(\theta_{5}+\theta_{6})+l_{4}\cos(\theta_{6}))\\ & +96(2l_{4}l_{3}\cos(\theta_{5})+l_{3}^{2}+l_{4}^{2}))+192l_{2}(l_{5}m_{6}\cos(\theta_{4}+\theta_{5}+\theta_{6})+l_{4}(m_{5}+2m_{6})\cos(\theta_{4}\\ & +\theta_{5})+l_{3}(m_{4}+2(m_{5}+m_{6}))\cos(\theta_{4}))+4l_{1}^{2}(m_{2}(\sin^{2}(2\theta_{1})\cos(2\theta_{2})+16)+48(m_{3}\\ & +m_{4}+m_{5}+m_{6}))+4l_{2}^{2}(m_{3}(\sin^{2}(2\theta_{1})\cos(2(\theta_{2}+\theta_{3}))+16)+48(m_{4}+m_{5}+m_{6}))\\ & +64l_{3}^{2}m_{4}+192l_{3}^{2}m_{5}+64l_{4}^{2}m_{5}) \end{array}$$

$$\begin{array}{ll} {\text{M}}_{23}={\text{M}}_{32}= & \frac{1}{192}(192l_{2}(l_{5}m_{6}\cos(\theta_{4}+\theta_{5}+\theta_{6})+l_{4}(m_{5}+2m_{6})\cos(\theta_{4}+\theta_{5})+l_{3}(m_{4}+2(m_{5}\\ & +m_{6}))\cos(\theta_{4}))+2l_{3}^{2}m_{4}\cos(2(\theta_{2}+\theta_{3}+\theta_{4}))-l_{3}^{2}m_{4}\cos(2(2\theta_{1}+\theta_{2}+\theta_{3}+\theta_{4}))\\ & -l_{3}^{2}m_{4}\cos(4\theta_{1}-2(\theta_{2}+\theta_{3}+\theta_{4}))+2l_{4}^{2}m_{5}\cos(2(\theta_{2}+\theta_{3}+\theta_{4}+\theta_{5}))-l_{4}^{2}m_{5}\cos(2(2\theta_{1}\\ & +\theta_{2}+\theta_{3}+\theta_{4}+\theta_{5}))-l_{4}^{2}m_{5}\cos(4\theta_{1}-2(\theta_{2}+\theta_{3}+\theta_{4}+\theta_{5}))+192l_{3}l_{4}m_{5}\cos(\theta_{5})\\ & +2m_{6}(l_{5}^{2}(32-(\cos(4\theta_{1})-1)\cos(2(\theta_{2}+\theta_{3}+\theta_{4}+\theta_{5}+\theta_{6})))+96l_{5}(l_{3}\cos(\theta_{5}+\theta_{6})\\ & +l_{4}\cos(\theta_{6}))+96(2l_{4}l_{3}\cos(\theta_{5})+l_{3}^{2}+l_{4}^{2}))+96l_{1}(l_{4}m_{5}\cos(\theta_{3}+\theta_{4}+\theta_{5})\\ & +m_{6}(2l_{4}\cos(\theta_{3}+\theta_{4}+\theta_{5})+l_{5}\cos(\theta_{3}+\theta_{4}+\theta_{5}+\theta_{6}))+l_{3}(m_{4}+2(m_{5}+m_{6}))\cos(\theta_{3}\\ & +\theta_{4})+l_{2}(m_{3}+2(m_{4}+m_{5}+m_{6}))\cos(\theta_{3}))+4l_{2}^{2}(m_{3}(\sin^{2}(2\theta_{1})\cos(2(\theta_{2}+\theta_{3}))+16)\\ & +48(m_{4}+m_{5}+m_{6}))+64l_{3}^{2}m_{4}+192l_{3}^{2}m_{5}+64l_{4}^{2}m_{5}) \end{array}$$

$$\begin{array}{ll} {\text{M}}_{24}={\text{M}}_{42}= & \frac{1}{192}(2l_{3}^{2}m_{4}\cos(2(\theta_{2}+\theta_{3}+\theta_{4}))-l_{3}^{2}m_{4}\cos(2(2\theta_{1}+\theta_{2}+\theta_{3}+\theta_{4}))-l_{3}^{2}m_{4}\cos(4\theta_{1}\\ & -2(\theta_{2}+\theta_{3}+\theta_{4}))+192l_{4}l_{3}m_{5}\cos(\theta_{5})+2l_{4}^{2}m_{5}\cos(2(\theta_{2}+\theta_{3}+\theta_{4}+\theta_{5}))\\ & -l_{4}^{2}m_{5}\cos(2(2\theta_{1}+\theta_{2}+\theta_{3}+\theta_{4}+\theta_{5}))-l_{4}^{2}m_{5}\cos(4\theta_{1}-2(\theta_{2}+\theta_{3}+\theta_{4}+\theta_{5}))\\ & +2m_{6}(l_{5}^{2}(32-(\cos(4\theta_{1})-1)\cos(2(\theta_{2}+\theta_{3}+\theta_{4}+\theta_{5}+\theta_{6})))+96l_{5}(l_{3}\cos(\theta_{5}+\theta_{6})\\ & +l_{4}\cos(\theta_{6}))+96(2l_{4}l_{3}\cos(\theta_{5})+l_{3}^{2}+l_{4}^{2}))+96l_{2}(l_{5}m_{6}\cos(\theta_{4}+\theta_{5}+\theta_{6})+l_{4}(m_{5}\\ & +2m_{6})\cos(\theta_{4}+\theta_{5})+l_{3}(m_{4}+2(m_{5}+m_{6}))\cos(\theta_{4}))+96l_{1}(l_{5}m_{6}\cos(\theta_{3}+\theta_{4}+\theta_{5}\\ & +\theta_{6})+l_{4}(m_{5}+2m_{6})\cos(\theta_{3}+\theta_{4}+\theta_{5})+l_{3}(m_{4}+2(m_{5}+m_{6}))\cos(\theta_{3}+\theta_{4}))+64l_{3}^{2}m_{4}\\ & +192l_{3}^{2}m_{5}+64l_{4}^{2}m_{5}) \end{array}$$

$$\begin{array}{ll} {\text{M}}_{25}={\text{M}}_{52}= & \frac{1}{96}(l_{4}m_{5}(48(l_{1}\cos(\theta_{3}+\theta_{4}+\theta_{5})+l_{2}\cos(\theta_{4}+\theta_{5})+l_{3}\cos(\theta_{5}))+l_{4}(32-(\cos(4\theta_{1})\\ & -1)\cos(2(\theta_{2}+\theta_{3}+\theta_{4}+\theta_{5}))))+m_{6}(l_{5}^{2}(32-(\cos(4\theta_{1})-1)\cos(2(\theta_{2}+\theta_{3}+\theta_{4}+\theta_{5}\\ & +\theta_{6})))+48l_{5}(l_{1}\cos(\theta_{3}+\theta_{4}+\theta_{5}+\theta_{6})+l_{2}\cos(\theta_{4}+\theta_{5}+\theta_{6})+l_{3}\cos(\theta_{5}+\theta_{6})\\ & +2l_{4}\cos(\theta_{6}))+96l_{4}(l_{1}\cos(\theta_{3}+\theta_{4}+\theta_{5})+l_{2}\cos(\theta_{4}+\theta_{5})+l_{3}\cos(\theta_{5})+l_{4}))) \end{array}$$

$$\begin{array}{ll} {\text{M}}_{26}={\text{M}}_{62}= & \frac{1}{96}l_{5}m_{6}(48(l_{1}\cos(\theta_{3}+\theta_{4}+\theta_{5}+\theta_{6})+l_{2}\cos(\theta_{4}+\theta_{5}+\theta_{6})+l_{3}\cos(\theta_{5}+\theta_{6})\\ & +l_{4}\cos(\theta_{6}))+l_{5}(32-(\cos(4\theta_{1})-1)\cos(2(\theta_{2}+\theta_{3}+\theta_{4}+\theta_{5}+\theta_{6})))) \end{array}$$

$$\begin{array}{ll} {\text{M}}_{33}= & \frac{1}{96}(96l_{2}(l_{5}m_{6}\cos(\theta_{4}+\theta_{5}+\theta_{6})+l_{4}(m_{5}+2m_{6})\cos(\theta_{4}+\theta_{5})+l_{3}(m_{4}+2(m_{5}+m_{6}))\cos(\theta_{4}))\\ & +l_{4}^{2}m_{5}(32-(\cos(4\theta_{1})-1)\cos(2(\theta_{2}+\theta_{3}+\theta_{4}+\theta_{5})))+m_{6}(96l_{5}l_{4}\cos(\theta_{6})+l_{5}^{2}(32\\ & -(\cos(4\theta_{1})-1)\cos(2(\theta_{2}+\theta_{3}+\theta_{4}+\theta_{5}+\theta_{6})))+96l_{4}^{2})+l_{3}^{2}(m_{4}(32-(\cos(4\theta_{1})\\ & -1)\cos(2(\theta_{2}+\theta_{3}+\theta_{4})))+96(m_{5}+m_{6}))+96l_{3}(l_{5}m_{6}\cos(\theta_{5}+\theta_{6})+l_{4}(m_{5}\\ & +2m_{6})\cos(\theta_{5}))+l_{2}^{2}(2m_{3}(\sin^{2}(2\theta_{1})\cos(2(\theta_{2}+\theta_{3}))+16)+96(m_{4}+m_{5}+m_{6}))) \end{array}$$

$$\begin{array}{ll} {\text{M}}_{34}={\text{M}}_{43}= & \frac{1}{96}(l_{3}^{2}(m_{4}(32-(\cos(4\theta_{1})-1)\cos(2(\theta_{2}+\theta_{3}+\theta_{4})))+96(m_{5}+m_{6}))+96l_{3}(l_{5}m_{6}\cos(\theta_{5}\\ & +\theta_{6})+l_{4}(m_{5}+2m_{6})\cos(\theta_{5}))+l_{4}^{2}m_{5}(32-(\cos(4\theta_{1})-1)\cos(\theta_{2}+\theta_{3}+\theta_{4}+\theta_{5})))\\ & +m_{6}(96l_{5}l_{4}\cos(\theta_{6})+l_{5}^{2}(32-(\cos(4\theta_{1})-1)\cos(2(\theta_{2}+\theta_{3}+\theta_{4}+\theta_{5}+\theta_{6})))+96l_{4}^{2})\\ & +48l_{2}(l_{5}m_{6}\cos(\theta_{4}+\theta_{5}+\theta_{6})+l_{4}(m_{5}+2m_{6})\cos(\theta_{4}+\theta_{5})+l_{3}(m_{4}+2(m_{5}\\ & +m_{6}))\cos(\theta_{4}))) \end{array}$$

$$\begin{array}{ll} {\text{M}}_{35}={\text{M}}_{53}= & \frac{1}{96}(l_{4}m_{5}(48l_{2}\cos(\theta_{4}+\theta_{5})+48l_{3}\cos(\theta_{5})+l_{4}(32-(\cos(4\theta_{1})-1)\cos(2(\theta_{2}+\theta_{3}+\theta_{4}\\ & +\theta_{5}))))+m_{6}(l_{5}^{2}(32-(\cos(4\theta_{1})-1)\cos(2(\theta_{2}+\theta_{3}+\theta_{4}+\theta_{5}+\theta_{6})))+48l_{5}(l_{2}\cos(\theta_{4}\\ & +\theta_{5}+\theta_{6})+l_{3}\cos(\theta_{5}+\theta_{6})+2l_{4}\cos(\theta_{6}))+96l_{4}(l_{2}\cos(\theta_{4}+\theta_{5})+l_{3}\cos(\theta_{5})+l_{4}))) \end{array}$$

$$\begin{array}{ll} {\text{M}}_{36}={\text{M}}_{63}= & \frac{1}{96}l_{5}m_{6}(48(l_{2}\cos(\theta_{4}+\theta_{5}+\theta_{6})+l_{3}\cos(\theta_{5}+\theta_{6})+l_{4}\cos(\theta_{6}))+l_{5}(32-(\cos(4\theta_{1})\\ & -1)\cos(2(\theta_{2}+\theta_{3}+\theta_{4}+\theta_{5}+\theta_{6})))) \end{array}$$

$$\begin{array}{l} {\text{M}}_{44}=\frac{1}{96}(l_{3}^{2}(m_{4}(32-(\cos(4\theta_{1})-1)\cos(2(\theta_{2}+\theta_{3}+\theta_{4})))+96(m_{5}+m_{6}))+96l_{3}(l_{5}m_{6}\cos(\theta_{5}+\\ \theta_{6})+l_{4}(m_{5}+2m_{6})\cos(\theta_{5}))+l_{4}^{2}m_{5}(32-(\cos(4\theta_{1})-1)\cos(2(\theta_{2}+\theta_{3}+\theta_{4}+\theta_{5})))+\\ m_{6}(96l_{5}l_{4}\cos(\theta_{6})+l_{5}^{2}(32-(\cos(4\theta_{1})-1)\cos(2(\theta_{2}+\theta_{3}+\theta_{4}+\theta_{5}+\theta_{6})))+96l_{4}^{2})) \end{array}$$

$$\begin{array}{ll} {\text{M}}_{45}={\text{M}}_{54}= & \frac{1}{96}(l_{4}m_{5}(48l_{3}\cos(\theta_{5})+l_{4}(32-(\cos(4\theta_{1})-1)\cos(2(\theta_{2}+\theta_{3}+\theta_{4}+\theta_{5}))))+m_{6}(l_{5}^{2}(32\\ & -(\cos(4\theta_{1})-1)\cos(2(\theta_{2}+\theta_{3}+\theta_{4}+\theta_{5}+\theta_{6})))+48l_{5}(l_{3}\cos(\theta_{5}+\theta_{6})+2l_{4}\cos(\theta_{6}))\\ & +96l_{4}(l_{3}\cos(\theta_{5})+l_{4}))) \end{array}$$

$$\begin{array}{ll} {\text{M}}_{46}={\text{M}}_{64}= & \frac{1}{96}l_{5}m_{6}(48l_{3}\cos(\theta_{5}+\theta_{6})+48l_{4}\cos(\theta_{6})+l_{5}(32-(\cos(4\theta_{1})-1)\cos(2(\theta_{2}+\theta_{3}+\theta_{4}\\ & +\theta_{5}+\theta_{6})))) \end{array}$$

$$\begin{array}{ll} {\text{M}}_{55}= & \frac{1}{96}(l_{4}^{2}m_{5}(32-(\cos(4\theta_{1})-1)\cos(2(\theta_{2}+\theta_{3}+\theta_{4}+\theta_{5})))+m_{6}(96l_{5}l_{4}\cos(\theta_{6})+l_{5}^{2}(32\\ & -(\cos(4\theta_{1})-1)\cos(2(\theta_{2}+\theta_{3}+\theta_{4}+\theta_{5}+\theta_{6})))+96l_{4}^{2})) \end{array}$$

$${\text{M}}_{56}={\text{M}}_{65}=\frac{1}{96}l_{5}m_{6}(48l_{4}\cos(\theta_{6})+l_{5}(32-(\cos(4\theta_{1})-1)\cos(2(\theta_{2}+\theta_{3}+\theta_{4}+\theta_{5}+\theta_{6}))))$$

$${\text{M}}_{66}=\frac{1}{96}l_{5}^{2}m_{6}(32-(\cos(4\theta_{1})-1)\cos(2(\theta_{2}+\theta_{3}+\theta_{4}+\theta_{5}+\theta_{6})))$$

The second term (the Coriolis and centrifugal forces term) and third term (gravitational effects term) of Equation (A27) can be calculated as follow:

(A28) $$V_{i}=\sum_{j=1}^{6}\sum_{k=1}^{6}\left(\frac{\partial M_{ij}}{\partial q_{k}}-\frac{1}{2}\frac{\partial M_{jk}}{\partial q_{i}}\right){\dot{q}}_{j}{\dot{q}}_{k}$$

(A29) $$G_{i}=-\sum_{j=1}^{6}m_{j}g^{T}J_{\upsilon j}^{i}$$
